# Cerebellar Ataxia With Neuropathy and Bilateral Vestibular Areflexia Syndrome Coexisting With JAK2-Positive Polycythemia Vera and Myelofibrosis

**DOI:** 10.31486/toj.24.0056

**Published:** 2025

**Authors:** Jayaram Saibaba, Jayachandran Selvaraj, Stalin Viswanathan, Vivekanandan Pillai

**Affiliations:** ^1^ Department of Neurology, Jawaharlal Institute of Postgraduate Medical Education and Research, Pondicherry, India; ^2^ Department of General Medicine, Jawaharlal Institute of Postgraduate Medical Education and Research, Pondicherry, India

**Keywords:** *Bilateral vestibulopathy*, *cerebellar ataxia*, *gait disorders–neurologic*, *neurodegenerative diseases*, *polycythemia vera*, *primary myelofibrosis*

## Abstract

**Background:**

Cerebellar ataxia with neuropathy and bilateral vestibular areflexia syndrome (CANVAS) is a rare, progressive, neurodegenerative disorder characterized by late-onset ataxia, bilateral vestibular impairment, and sensory neuropathy.

**Case Report:**

A 51-year-old male presented to the hospital with worsening dizziness, tremulousness of limbs, and falls during the preceding year. The patient experienced gradually progressive sensorimotor lower motor neuron quadriparesis, asymmetric ataxia, chronic pancerebellar dysfunction, oscillopsia, and impaired vestibulo-ocular reflex. His comorbidities included poorly controlled type 2 diabetes mellitus, chronic alcohol use, and thalidomide therapy for polycythemia vera with myelofibrosis. Diagnostic workup revealed sensory axonal neuropathy, hypercellular bone marrow with myelofibrosis, and utriculo-saccular dysfunction. Diabetes and thalidomide- and alcohol-related complications were presumed to be the reason for the patient's symptoms, but investigations revealed a diagnosis of CANVAS coexisting with polycythemia vera. The patient was treated with rehabilitation exercises and medications that slightly improved but did not resolve his symptoms. More than 1 year after the patient's last follow-up, a physician at another hospital discontinued the thalidomide prescription because of the patient's neuropathy. Two months later, the patient developed febrile neutropenia and died of pneumonia and sepsis.

**Conclusion:**

To our knowledge, CANVAS coexisting with polycythemia vera has only been reported once in the literature. The significance of this coexistence is not clear. Future case studies may help elucidate a link between these two entities.

## INTRODUCTION

Cerebellar ataxia with neuropathy and bilateral vestibular areflexia syndrome (CANVAS) is a progressive neurologic disorder that can be either familial or sporadic.^1^ The acronym CANVAS debuted in 2011, and the syndrome has been documented primarily through case reports.^2^ A recessive AAGGG repeat expansion in intron 2 of replication factor complex subunit 1 (RFC1) was discovered in 2019 in patients with familial CANVAS.^3^ CANVAS occurs predominantly in the sixth decade, and the complete triad of cerebellar dysfunction, bilateral vestibular hypofunction, and somatosensory deficit is seen in two-thirds of patients.^2^ Development of the full triad may take more than 10 years.^4^ The manifestations of cerebellar dysfunction include speech disorders, gait ataxia, dysphagia, and disturbance in movement coordination.^4^

Polycythemia vera is a chronic myeloproliferative disorder characterized by a somatic mutation affecting JAK2 cytokine receptors that leads to the abnormal proliferation of myeloid hematopoietic cells. Polycythemia vera can result in extramedullary hematopoiesis, prothrombotic complications, blast transformation, or myelofibrosis. The incidence of polycythemia vera in the United States is 0.8 to 1.3 per 100,000 population.^5^ To our knowledge, polycythemia vera coexisting with CANVAS has only been reported in one published case.^6^

## CASE REPORT

A 51-year-old male crane operator from South India returned from Saudi Arabia in 2014 because of his inability to walk independently and operate the crane. He presented to the hospital in 2020 with worsening dizziness, tremulousness of limbs, and falls during the preceding year. For the prior 6 years, the patient had complained of vertigo and difficulty walking on the streets alone. Four years prior, he developed weakness of his left-sided limbs, followed by weakness of his limbs on the right side. Initially, the left upper limb weakness was predominantly distal, whereas the lower limb weakness was distal and proximal. The patient's symptoms had increased during the prior year. He could not grip footwear, could not feel the socks on his feet, and had increased sweating of the feet compared to his usual state of health. During the prior year, the patient also experienced blurred vision, oscillopsia, worsening dizziness on turning his head to either side, slurred speech, and tremulousness in both upper limbs. He had fallen 3 times while walking at home and was only able to walk with his eyes looking downward. He also complained of rapid fatigability.

The patient had had type 2 diabetes mellitus for the prior 10 years that was well-controlled with metformin 500 mg twice daily (HbA1c of 6.5%). He had been diagnosed with JAK2-positive polycythemia vera in 2014, a few months after his neurologic symptoms began. Four years after the diagnosis of polycythemia vera and 2 years prior to presentation, the patient was diagnosed with grade 2 secondary myelofibrosis and was treated with thalidomide (100 mg once daily) and prednisolone (25 mg once daily) for 7 months (until December 2018) when treatment was discontinued because of financial constraints. Thalidomide was restarted 4 months later (April 2019), and the patient remained compliant. He regularly consumed alcohol for 5 years (150 g per day) but stopped after he developed dizziness. He had a 15 pack-year history of smoking, but he quit smoking after the diagnosis of polycythemia vera.

At admission, the patient was conscious, oriented, and afebrile, with a pulse rate of 78 beats per minute, blood pressure of 100/70 mm Hg, respiratory rate of 18 breaths per minute, and oxygen saturation of 99% on room air. Abdominal examination revealed splenomegaly of 8 cm below the subcostal margin. Grade 3 bilateral nystagmus and broken pursuits were observed. Fundus examination was normal. Muscle strength was 4+/5 in the proximal upper limbs and lower limbs and 4-/5 in the distal upper limbs and lower limbs. All reflexes were brisk except for an ankle jerk of ±1, and the plantar reflexes were flexors. On sensory examination, pain, temperature, and fine and crude touch were intact; however, joint position and vibration sensations were impaired. The patient had left-sided dysdiadochokinesia, impaired past pointing, and an abnormal heel-knee-shin test. Gait and stance ataxia were also observed. The patient swayed to the right during the Romberg test. Head impulse test for vestibulo-ocular reflex was abnormal bilaterally.

Given the insidious onset, gradually progressive sensorimotor quadriparesis, pancerebellar dysfunction, and impaired joint position sense, a diagnosis of chronic progressive cerebellar ataxia and peripheral neuropathy from chronic degenerative processes or genetic causes—with additional contribution by diabetes, thalidomide, and alcohol—was considered.

**Figure 1.**
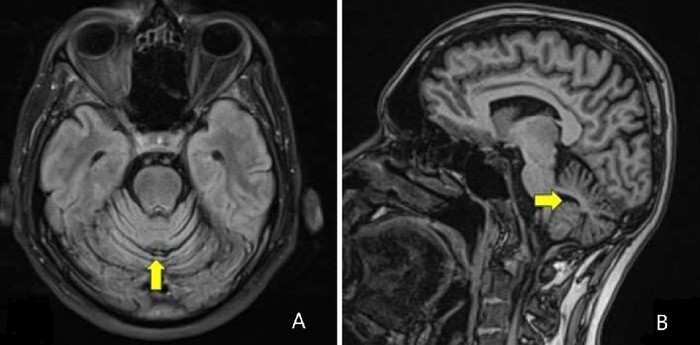
(A) Axial magnetic resonance imaging (MRI) of the brain shows gross cerebellar atrophy (arrow). (B) Sagittal MRI of the brain shows dorsal and anterior vermian and paravermian cerebellar atrophy (arrow).

**Figure 2.**
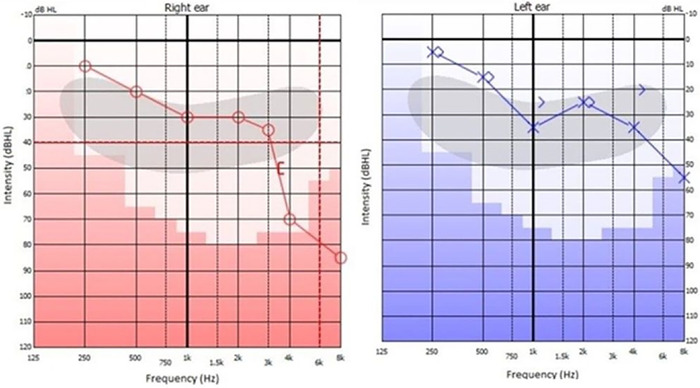
**Pure tone audiometry shows bilateral mild sloping sensorineural hearing loss.** dBHL, decibel hearing level; Hz, hertz.

Magnetic resonance imaging (MRI) of the brain showed vermian and paravermian cerebellar atrophy (Figure 1). Nerve conduction study revealed bilateral sensory axonal neuropathy with normal motor conduction velocities. Audiometry revealed mild bilateral sensorineural hearing loss (Figure 2). Immittance tympanometry showed type A tympanogram bilaterally. Abnormal vestibular-evoked myogenic potentials interaural amplitude asymmetric ratio of 48.66% was observed, suggesting bilateral utriculo-saccular dysfunction.

The patient's erythrocyte sedimentation rate was normal. Bone marrow showed hypercellular marrow with erythroid and megakaryocytic hyperplasia, 5% blasts, and World Health Organization grade 1 myelofibrosis. Cerebrospinal fluid (CSF) was acellular, with normal biochemistry and sterile cultures, and was negative for acid-fast bacilli, syphilis, cryptococci, and malignancy. The CSF paraneoplastic panel (anti-Hu, anti-Ri, anti-Yo, anti-CV2, anti-PNMA2, anti-amphiphysin, anti-PCA-2, anti-SOX2, anti-Tr, anti-GAD65, and anti-myelin) was negative. Contrast-enhanced computed tomography of the thorax and abdomen revealed no masses except splenomegaly.

Medical Oncology was consulted, and the continuation of 100 mg of thalidomide once daily at bedtime was advised. The patient was treated symptomatically with vestibular rehabilitation exercises (head and eye movements while lying down, sitting, standing, and walking) and was discharged after 16 days of hospitalization with oral antidiabetic drugs (metformin 500 mg twice daily, glimepiride 1 mg twice daily), amitriptyline (25 mg once daily at night for paresthesias that were probably diabetes-related), and thalidomide 100 mg once daily.

From 2020 onwards, the patient continued taking these medications. Because of coronavirus disease 2019–related travel restrictions, he followed up again only in 2022, 1.5 years postdischarge. He could walk with support without falling at home but reported no improvement in his dizziness, fatigue, or imbalance. Seventeen months after his last follow-up with us (in August 2023), a physician at another hospital discontinued the thalidomide because of the patient's neuropathy. Two months after the patient visited the outside physician, he developed febrile neutropenia and died of pneumonia and sepsis.

## DISCUSSION

CANVAS results from the impairment of cerebellar, vestibular, and sensory functions, leading to a progressive and severe balance impairment. The initial report on the syndrome described late-onset ataxia in 4 patients with bilateral vestibular deficits, cerebellar dysfunction, and impaired vestibulo-ocular reflex but did not report peripheral neuropathy.^7^ Sensory axonal neuropathy is now included as a fundamental component of CANVAS.

In a study by Cortese et al, among 100 genetically proven cases of CANVAS, imbalance was the presenting symptom in 50% of patients, and autonomic dysfunction was observed in 50% of the 42 patients who were tested, while 32 had symptoms.^2^ Thirty-two patients had oscillopsia, and sensory symptoms were seen in 76 patients.^2^ Chronic cough was also observed in 64 patients,^2^ but our patient did not have this symptom.

In a series of 80 patients, Szmulewicz et al suspected CANVAS in patients who presented with the triad of cerebellar dysfunction, vestibulopathy, and somatosensory dysfunction.^4^ However, because development of all 3 features may take 10 years or more, the authors recommend that patients with 2 features of the triad should have baseline investigations for CANVAS after ruling out other possible causes, such as spinocerebellar ataxia (SCA), and then should be followed at regular intervals.^4^ Szmulewicz et al recommend that patients with suspected CANVAS be screened for SCA types 1, 2, 3, 6, and 7. SCA-3 is associated with vestibulopathy and neuropathy.^4^

Other close differentials for our patient's symptoms are Friedreich ataxia and the olivopontocerebellar variant of multiple system atrophy.^7^ Although Friedreich ataxia is a CANVAS mimic, it has a young onset (second to third decade) and is associated with muscle wasting and skeletal deformities.^8^ Also, our patient had type 2 diabetes mellitus before the onset of CANVAS, while in patients with Friedreich ataxia, diabetes occurs later. Multiple system atrophy can be identified by the absence of sensory neuropathy and vestibulopathy with characteristic signs on MRI imaging.^9^ We did not perform genetic testing (polymerase chain reaction and Southern blot) for SCA and Friedreich ataxia because of the lack of such facilities at our institution.

**Figure 3.**
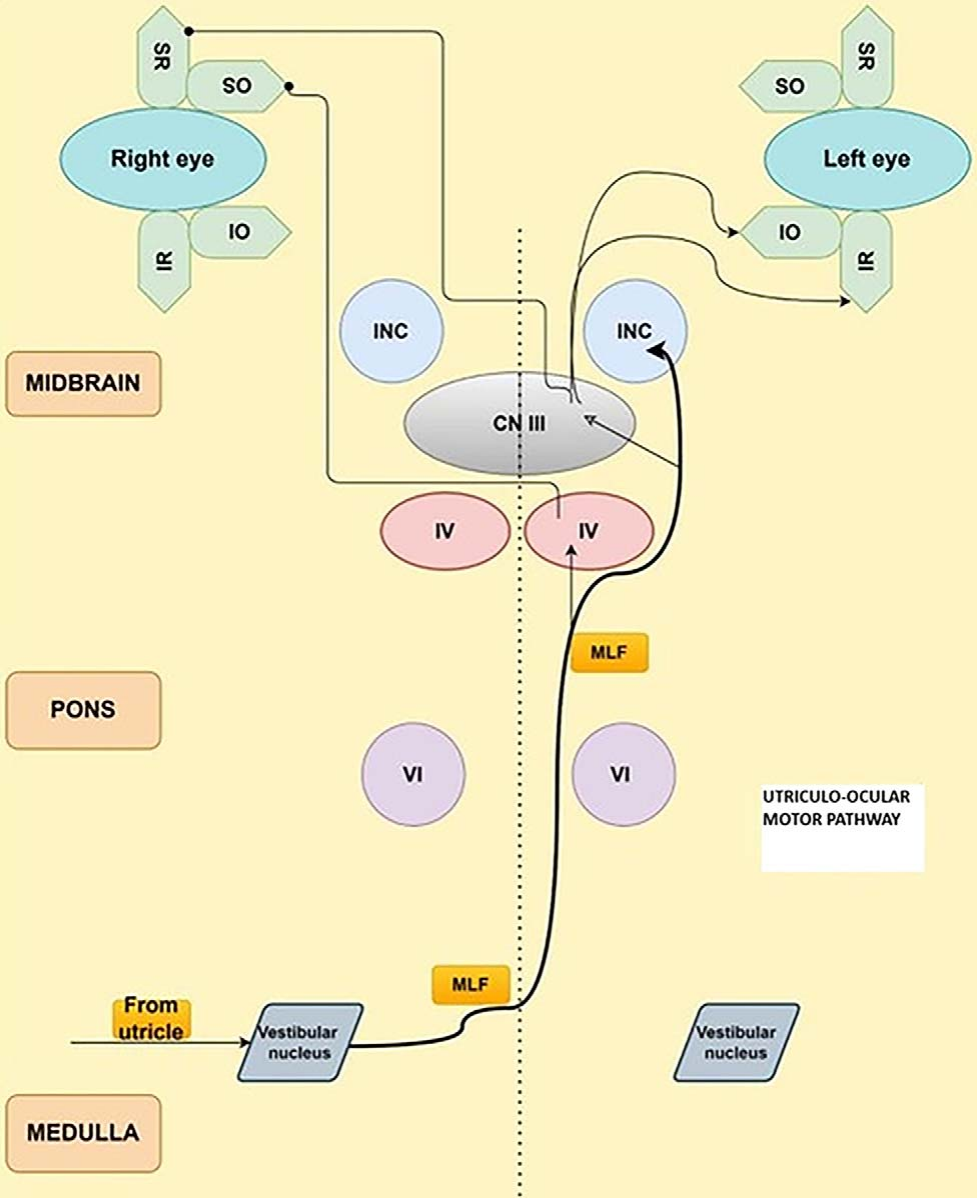
**Schematic diagram of the utriculo-ocular motor pathway. Lesions in both vestibular nerves and utriculo-saccular dysfunction cause an impaired vestibulo-ocular reflex. Vestibular dysfunction is one component of the triad in cerebellar ataxia with neuropathy and bilateral vestibular areflexia syndrome (CANVAS).** CN III, cranial nerve III, oculomotor nerve; [CN] IV, trochlear nerve; [CN] VI, abducens nerve; INC, interstitial nucleus of Cajal; IO, inferior oblique; IR, inferior rectus; MLF, medial longitudinal fasciculus; SO, superior oblique; SR, superior rectus. Adapted with permission of the creator Daniel R. Gold, DO from the Neuroophthalmology Virtual Education Library: NOVEL (online) available at novel.utah.edu/Gold/.

The vestibular and visual reflexes stabilize the normal gaze during activity (Figure 3). The optokinetic reflex (that stabilizes an entire scene oscillating side to side), smooth pursuit movement (that stabilizes a small target moving side to side), and vestibulo-ocular reflex (that stabilizes the gaze during head movement) contribute to the visually enhanced vestibulo-ocular reflex.^7^ An abnormal visually enhanced vestibulo-ocular reflex suggests combined vestibular and cerebellar dysfunction. Impairment of this reflex may be visible at the bedside in patients with CANVAS.^10^ Recent (2021) pathologic evidence of CANVAS based on temporal bone biopsies points to a vestibular ganglionopathy (loss of Scarpa cells) and atrophy of the vestibular nerves^11^ instead of the vestibular nuclei, as proposed earlier (2004) by Migliaccio et al.^7^ Vestibular ganglionopathy is similar to the involvement of the facial and trigeminal nerves in CANVAS, which is often inapparent or clinically asymptomatic and detected only by electrophysiology or histopathology.^11^ Patients with Friedreich ataxia and CANVAS have vestibular ganglion atrophy, while the vestibular nuclei are involved in patients with SCA-3.^11^

Our patient's comorbid conditions of type 2 diabetes mellitus, alcohol use, polycythemia vera, and thalidomide therapy do not cause CANVAS but exacerbate the neurodegenerative changes—such as polyneuropathy, autonomic dysfunction, and cerebellar dysfunction—of the syndrome. The significance of coexisting polycythemia vera and CANVAS in our patient is not clear. The progression of polycythemia vera and secondary complications contributed to our patient's poor prognosis.

Management of CANVAS includes vestibular rehabilitation, prevention of falls, and treatment of pain and autonomic dysfunction. The patient's vertigo had not improved with exercises when he followed up 1.5 years postdischarge. However, his falls did not occur when he used sandals and a cane or attendant at home. The patient's pain was controlled with amitriptyline.

## CONCLUSION

CANVAS is a rare, progressive, neurodegenerative disorder that closely mimics SCA-3, Friedreich ataxia, and multiple system atrophy. Supportive treatment and vestibular rehabilitation exercises may somewhat improve the quality of life of patients with CANVAS. Type 2 diabetes mellitus, alcohol use, and thalidomide confounded the initial clinical picture in our patient. Polycythemia vera coexisting with CANVAS in this patient was an unusual presentation, and to our knowledge, our case is only the second report of this concurrence. Future case studies may help elucidate a link between these two entities.

## Notes

*This article meets the Accreditation Council for Graduate Medical Education and the American Board of Medical Specialties Maintenance of Certification competencies for Patient Care and Medical Knowledge.*
